# Collaborative reasoning in the context of group competition

**DOI:** 10.1371/journal.pone.0246589

**Published:** 2021-02-05

**Authors:** Andreas Domberg, Michael Tomasello, Bahar Köymen

**Affiliations:** 1 Max Planck Research Group iSearch, Max Planck Institute for Human Development, Berlin, Germany; 2 Department of Psychology and Neuroscience, Duke University, Durham, North Carolina, United States of America; 3 Department of Developmental and Comparative Psychology, Max Planck Institute for Evolutionary Anthropology, Leipzig, Germany; 4 School of Health Sciences, University of Manchester, Manchester, United Kingdom; Middlesex University, UNITED KINGDOM

## Abstract

A key skill in collaborative problem-solving is to communicate and evaluate reasons for proposals to arrive at the decision benefiting all group members. Although it is well-documented that collaborative contexts facilitate young children’s reasoning, less is known about whether competition with other groups contributes to children’s collaborative reasoning. We investigated whether between-group competition facilitates children’s within-group collaborative reasoning, regarding their production of reasons and their use of *transacts*, communicative acts that operate on one another’s proposals and reasoning. We presented 5- and 7-year-old peer dyads with two collaborative problem-solving tasks (decorating a zoo and a dollhouse). In one task, children competed against another group (the competitive condition); whereas in the other task, they did not (non-competitive condition). Our results suggest that children’s sensitivity to group competition as reflected in their reasoning changed depending on the task. When they decorated a house, they produced more transacts in the competitive condition than in the non-competitive condition; whereas when they decorated a zoo, this pattern was reversed. Thus, our results highlight that group competition did not influence children’s collaborative reasoning consistently across different contexts.

## Introduction

A key step in collaborative problem-solving is communicating and evaluating reasons provided by the individuals working together [1,2]. Humans excel at this kind of reasoning in groups and this ability of sharing reasons to make rational collaborative decisions contributes to humans’ evolutionary success [3]. It confers advantages in dealing with challenges in the physical and social environment, as well as in group competition. Competition between groups has been found to increase motivation for in-group collaborative activities. Studies with adults and children report that individuals contribute more to collaborative group activities when their group competes with another group [4–6] (but see [7]).

Young children’s ability to collaboratively solve problems or their collaborative reasoning has been well documented (see [6,8,9] for reviews). When working towards a joint goal, children as young as age 3 take into account their common ground with their peer partners and provide reasons to convince them only when they expect them to be naïve or unaware of certain facts [10] or adjust the informativeness of reasons depending on how much common knowledge they can expect in their partners [11,12]. In pursuing a joint goal, preschool children not only produce reasons to justify their own proposals for their partners, but they also evaluate the proposals and the reasoning of their partners, referred to as *transactive statements* or *transacts* [13–17]. Through these transacts, children revise their beliefs if their partner presents a conflicting belief supported by better reasons [18] and prefer to reason with partners who submit to good reasons [19].

It has also been documented that the context of collaboration, as opposed to competition, facilitates children’s reasoning. In a number of studies, when children competed against each other (where reasoning served to determine who wins and who loses), as opposed to collaborating (where reasoning served to help both parties win together), their reasoning suffered [20–22]. For instance, Domberg and colleagues [20], presented 5- and 7-year-old dyads with a task of building a zoo together. Half of the dyads played the game cooperatively (if they place the toy animals in the right places, each child would get a reward); whereas the other half of the dyads played the game competitively (only the child who has more toy animals on their side would get a reward). Both age groups produced more reasons, and also more complex reasons, in the cooperative condition than in the competitive condition.

Nonetheless, competition has also been observed to lead to successful outcomes, particularly when the competition is between groups, because it fosters in-group collaboration [3]. Specifically, competing with another group might enhance the motivation for making right collaborative decisions. In fact, in educational contexts, group competition has been shown to engage children and motivate better outcomes [23,24]. For instance, two programs that structure the classroom as a competition between student groups while also emphasizing individuals’ accountability towards their own group have been shown to facilitate greater post-manipulation achievement gains than purely cooperative, competitive or traditional instruction methods. However, beyond this educational context, research on influences from group competition remains scant. Thus, our question in the present study is whether group competition has an impact on children’s collaborative reasoning, and how this impact changes at different developmental stages.

In the current study, we gave 5- and 7-year-old peer dyads two cooperative problem-solving tasks, decorating a zoo and a dollhouse by adding, removing, and replacing items (tokens with pictures on them). The dyads carried out one task in the competitive condition in which the dyads competed against another dyad (they saw an image of another pair playing the same game, without seeing their decisions), and were instructed that they would win only if they did a better job than the other team. The dyads carried out the other task in the non-competitive condition in which the dyads simply collaborated with each other without competing against another team, and were instructed that they would win only if they did a good job. We hypothesized that in the competitive condition, as compared to the non-competitive condition, children would spend greater effort on the task, producing more reasons and being more critical about one another’s proposals and their reasons for these proposals, so they would produce more transacts for each item. We selected these age groups because around age 5, children have been shown to begin to understand (and adapt their reasoning to) cooperative and competitive contexts; whereas 7-year-olds’ sensitivity to competition is more pronounced [20] and at this age, particularly effective group reasoning has been shown [25].

## Methods

### Participants

Sixty-four 5-year-olds (*M* = 5;9, *SD* = 2 months, 32 girls) and 64 7-year-olds (*M* = 7;6, *SD* = 4 months, 32 girls) in 64 same-age and same-sex dyads participated in this study. The sample size was determined prior to data collection and was based on prior work that had similar research questions and used similar methodologies [13]. The study took place in children’s nurseries and schools. Each dyad participated in both conditions. All children were native German speakers with various socioeconomic backgrounds. The study was approved by the Max Planck Institute for Evolutionary Anthropology Child Subjects Committee. Written consent was received from the children’s parents, and in accordance with all applicable laws and rules governing psychological research in Germany.

### Materials

The materials for each round (warm-up and two test trials) consisted of a background picture, a set of “default” items already placed on it, and two sets of further items, one per child. The number of items that each child received was twice the number of default items, with half of them always relevant to the respective background picture and the other half irrelevant. We added this aspect of relevance, and for some items redundancy, to give children some points to use in reasoning. Children always first had to discard half of their items individually before discussing together which of the remaining items stay in the picture to ensure that each child would start the discussion with a set of items that they had thoroughly reviewed and personally approved of.

In the warm-up, the background picture was of a picnic basket with five default items. Each child received ten items so as to later start the discussion with their selection of five of them. In the test trials, children played the zoo game in one condition and the dollhouse game in the other condition. The background picture in each game included seven default items. Each child individually received 14 items and was asked to select seven (see S1 File in the Supporting Materials for a listing of the complete set of materials). Again, out of the seven relevant items that each child received, some were redundant across children for them to have straightforward points to discuss about.

#### Procedure

Children sat down at a table facing each other. During the warm-up phase instructed by the experimenter (E), children played a game that mirrored the rules and structure of the games in the two critical trials. The goal of the warm-up game was to decorate, in the best way, the picnic basket picture background that already included the five default items to be replaced or kept. E asked children what things they associate with picnics. E then introduced the picnic background. Each child received ten items for decorating the picnic basket. Each child individually was asked to select five of their own items that they thought were adequate and discard five items that they deemed unsuitable. Then, children were asked to decide together on which five of the ten shortlisted items should and should not end up in the picnic basket. E involved children in a guided discussion aimed at production of reasons for each decision and mutual consensus among children about which items make for a good picnic and should thus be included. Specifically, E asked what items children wanted to act on, why, and to the respective other child, whether the reason given was good, until children signalled that they were done. Upon that, E commended the final result in the warm-up phase and removed the warm-up materials.

In the two critical trials, we gave children the zoo and the dollhouse game in turn, with the task being analogous to the warm-up. Also, children made their individual choices privately with blinds between them to shift any discussions about coordinating their selections into the actual test phase.

When children played the zoo game in the competitive condition, upon children’s individual selection of seven items each, E presented the zoo background with seven default items, explaining, “You need to put a really nice zoo together. Think about how many of these items you want to replace. But make sure you always give a good reason for why you want to replace them.” E opened a laptop and said, “Let me see if you are playing this one against another team. Here’s the red team! They are already done decorating their zoo. But if you end up having a nicer zoo than the red team, then you will win!” E left the children alone for their discussion, which was video recorded. Once children called E back, E removed the zoo materials, without commenting on their choices. Then E introduced the dollhouse game to be played in the non-competitive condition. The procedure was identical to the competitive condition, except that E told children that they were not going to compete with another group, “Let me see if you are playing against someone else. No, this round you’ll be playing alone, there’s no-one else. If you end up having a really nice house, then you will win!”.

The order of conditions and the game played in each condition were counterbalanced across dyads. See also S2 File in the Supporting Materials for the test protocol in English.

### Coding

Children’s conversations were transcribed verbatim with each line in the transcript corresponding to one proposition. First, we did a manipulation check that served to ensure children were aware of their group-competitive situation in the competitive condition. We coded if they a) attended to, or commented on the red team during the trial, or b) expressed a competitive group motivation when introduced to the red team. For instance, some children would physically display rivalry towards the laptop.

Next, we identified reasons in children’s talk, which were any kind of explanation for or against selecting or discarding an item. Finally, we identified *transacts*, which were defined as cases of one child commenting on their partner’s proposals and reasons. Then we calculated the number of items, out of 21 items (seven shortlisted items by each child and the default seven items available in the background), for which children produced transacts. In other words, we calculated the number of items (out of 21) that children jointly attended to and mutually tried to propose or justify acting on. A second coder, who was blind to conditions and predictions of this study coded the transcripts of eight dyads (four in each age group, 12.5% of the total sample) for the identification of arguments and transacts. The agreement was *κ* = .80 and *κ* = .87 respectively. Also, a repeated manipulation check by this second coder on 16 competitive trials (eight per age group, 25% of the total sample) reached 100% agreement.

## Results

Our manipulation check indicates that 27 out of 32 5-year-old dyads and 26 out of 32 7-year-old dyads mentioned or attended to the competitor group in the competitive condition. When we compared the number of dyads who mentioned the other group in their conversations to chance using binomial tests, this number was significantly above chance for each age group (*p* < .001 in both age groups). We can thus conclude that the condition manipulation was successful and go on to analyze the transcripts.

**Fig 1:**
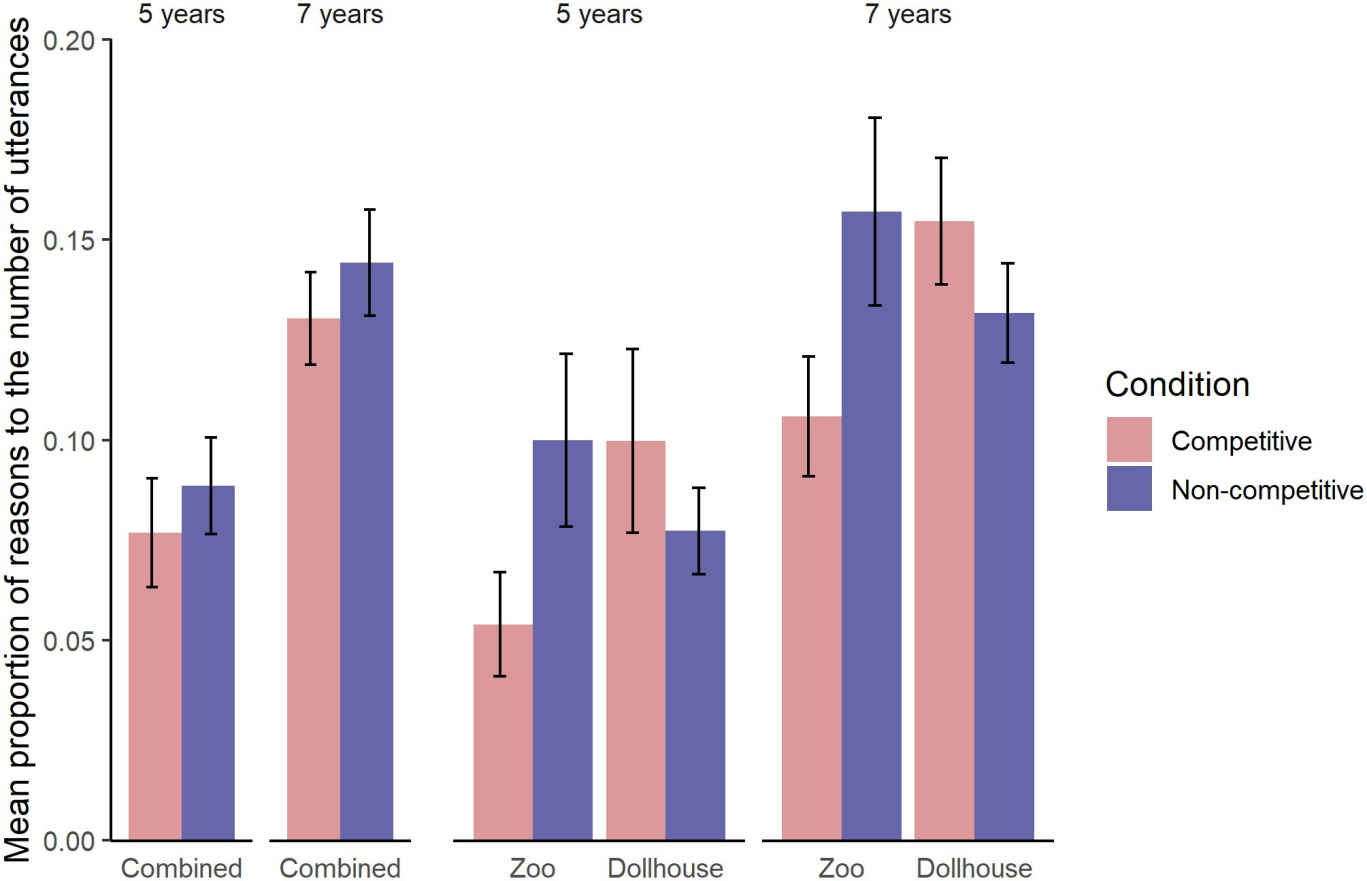
The mean frequency of reasons per utterance for each age group, condition and game-condition assignment.

As part of the main analyses, we first investigated whether the frequency of reasons changed across conditions and age groups. Out of 6,157 utterances that children produced, 753 were coded as reasons. We fitted a Generalized Linear Mixed Model (GLMM) with Poisson error distribution. The response variable was the number of reasons that children produced in each condition. Predictors in the full model were age group (5, 7), condition (competitive, non-competitive) and their interaction, along with the control variables: game (zoo, dollhouse), gender, order of conditions (first, second); as well as a random intercept for each dyad, as we had two observations per dyad (one in each condition). We also added the number of utterances per trial as an offset term to account for dyads’ varying talkativeness. We compared this model to a null model that included only the control variables, random intercept, and the offset term. The fit of the full model compared to the null model was significantly better (*χ*^*2*^ = 12.52, *df* = 3, *p* = .006). To test the significance of the interaction term between age group and condition, we compared the full model with a reduced model that only lacks the interaction term and this comparison revealed that the interaction was not significant (*χ*^*2*^ = 0.08, *df* = 1, *p* = .78). Thus, we dropped the interaction term to make the main effects interpretable, and compared the resulting reduced model containing main effects of age group and condition with two models that lack either of these predictors respectively. These comparisons suggested that there was no significant main effect of condition (*χ*^*2*^ = 1.92, *df* = 1, *p* = .17), but a significant main effect of age (*χ*^*2*^ = 10.47, *df* = 1, *p* = .001). Thus, there was no significant difference in the frequency of the reasons between the competitive and non-competitive condition; and 7-year-olds produced reasons more frequently in their discussions than did 5-year-olds (see Fig 1). See also S1a Table in the Supporting Materials for a summary of this model.

Upon settling our main experimental hypothesis by finding no effect of our condition manipulation, a closer inspection of the data suggested an interaction of condition with game. Therefore, our exploratory analysis focused on re-running the reduced main effects model above and including the 2-way interaction term between condition and game. This model significantly improved the fit over the main effects model, suggesting a significant interaction of condition and game (*χ*^*2*^ = 6.89, *df* = 1, *p* = .009; see S1b Table in the Supporting Materials for a summary of this model). That is, when children played the dollhouse game, they produced more reasons in the competitive condition than in the non-competitive condition (*χ*^*2*^ = 8.81, *df* = 1, *p* = .003); whereas when they played the zoo game, they produced more reasons in the non-competitive condition than in the competitive condition (*χ*^*2*^ = 19.83, *df* = 1, *p* < .001, see Fig 1 and S4 File). It should be noted that the pattern was the same across age groups (see Fig 1 and S4 File).

**Fig 2:**
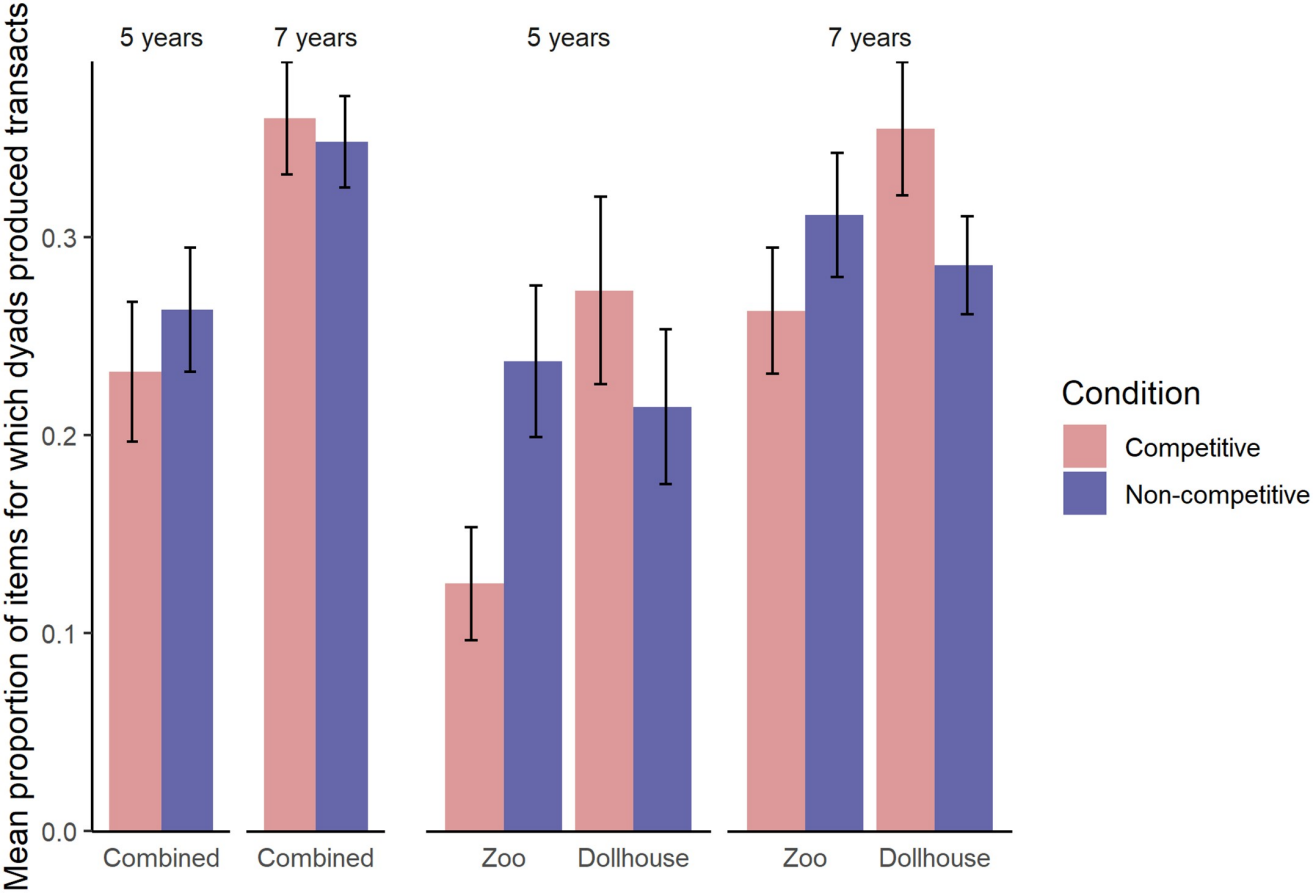
The mean proportion of transacts produced by each dyad for each age group, condition and game-condition assignment.

Next, we investigated whether the likelihood of the dyads’ production of transacts varied across conditions and age groups, using a GLMM with binomial error distribution. The unit of analysis was each of the 21 items, the response variable was the binomial measure of whether a dyad produced transacts for this item, and the predictors in the full and null model were the same as in the full-null comparison of our main analysis above (both models: gender, game, order of conditions; full model: age, condition and their interaction). Again, the full-null model comparison indicated that the full model fitted better (*χ*^*2*^ = 12.83, *df* = 3, *p* = .005). To test the significance of the interaction term between age group and condition, we compared the full model with a reduced model that only lacks the interaction term and this comparison revealed that the interaction was not significant (*χ*^*2*^ = 1.61, *df* = 1, *p* = .20). Thus, we dropped the interaction term to make any main effects interpretable, and compared the resulting reduced model containing main effects of age group and condition with two models that lack either of these predictors respectively. These comparisons suggested that there was no significant main effect of condition (*χ*^*2*^ = .33, *df* = 1, *p* = .57), but a significant main effect of age (*χ*^*2*^ = 10.89, *df* = 1, *p* < .001). Thus, the likelihood of producing transacts for each item did not vary significantly across the competitive condition and the non-competitive condition; and 7-year-olds were more likely to produce transacts about items than were 5-year-olds (Fig 2). See also S1c Table in the Supporting Materials for a model summary.

In our exploratory analysis, comparing the main effects model to a model that contains the interaction term between condition and game, we again found that this latter model showed an improved fit to the data (*χ*^*2*^ = 6.67, *df* = 1, *p* = .010), suggesting an interaction effect between condition and game (see S1d Table in the Supporting Materials for a model summary). When children played the dollhouse game, they were more likely to produce transacts about items in the competitive condition than in the non-competitive condition (*χ*^*2*^ = 5.67, *df* = 1, *p* = .017); whereas this pattern was reversed when children played the zoo game (*χ*^*2*^ = 19.83, *df* = 1, *p* < .001; see Fig 2 and S4 File).

## Discussion

This study aimed to elucidate whether outgroup competition can trigger better reasoning among collaborating children, specifically whether it can motivate more actions being discussed and more reasons being brought up for and against the actions under consideration. Our results suggest that there is no significant effect of outgroup competition upon the extent of children’s reasoning. One explanation for this could be that children in the competitive condition may not have perceived the game as a group competition. The manipulation check, however, suggests that children were indeed aware of the other team, commenting about them and showing group-directed affect in their peer conversations. Thus, failing to notice the other group is unlikely to explain this absence of a clear effect of group competition.

A second, more plausible, explanation for the non-significant effect of outgroup competition could be the similarity between conditions. To make the two conditions comparable, we took a conservative approach. In both conditions, we introduced a joint goal of winning (winning against another team in the competitive condition vs. winning the game on their own in the non-competitive condition). Therefore, the non-competitive condition was perhaps too motivating. Conversely, a more immediate operationalization of competition, e.g., by having dyads compete directly against each other while having them complete their tasks in the same room, could have perhaps made the competitive condition more motivating.

Third, as mentioned earlier, there have been studies both with adults [4,5] and children [26] that showed effects from outgroup competition. For instance, in a monetary Public Goods Game [5] group members could contribute endowed money to a group pot that was then multiplied by a factor and paid out evenly. The study by [26] had the multiplication and pay-out replaced by an immaterial reward in the form of the group’s rank in a tournament. In both paradigms, participants are faced with the dilemma of whether to share a finite resource with their team for some form of group-level success, or to keep their endowment; and in both studies, out-group competition led to increased in-group sharing (see also [27,28]).

Potential reasons no such effect was observed in our study are twofold but related: First, joint reasoning as a measure does not face participants with the exact same dilemma of sharing or keeping a finite resource. Rather, participants’ “risk” or cost consisted in expending greater reasoning effort without attaining a better outcome. Second, recall that children in the present study were tasked not with producing as many reasons or discussing as many items as possible, but with making good decisions. Unlike typically with material prosocial contributions, in the case of reason-giving, more of the same does not straightforwardly lead to better group outcomes (see also [29] for a related empirical finding with adult discussion groups). And to judge whether their current outcome is good enough, children lacked concrete feedback as to whether they were making good decisions. These are, however, necessary conditions if the motivation to make better decisions than the other group is supposed to drive a response that adapts to that group. So in summary, if the number of reasons produced in joint reasoning relates to the degree of group commitment, it works based on mechanisms other than the finiteness of an endowed resource, as has been involved in the above studies [7].

However, this does not mean it is impossible for competition to influence the production of reasons. In a study contrasting cooperating peers’ discussions to those between competing peers, peers who were collaborating produced more reasons than peers who were competing [20]. The mechanism that likely modulated the number and kind of reasons produced was omission: In one competitive condition (Study 2), one child learned a set of reasons prior to discussing a joint decision with a peer. The outcome of the peer discussion would decide about who gets a reward, and the learned reasons would favour the other child. Children in that situation tended to withhold the reasons that they had learned. In contrast, in the competitive condition of the present study, in which the competing groups did not interact with each other directly, withholding reasons was irrelevant, as producing a reason could not worsen a dyad’s chances of winning, and similarly withholding a reason could not improve those chances. In summary, strategic omission of reasons or evidence can be a tool in reasoning with competitors directly, but not within a cooperative group discussion. Consequently, this mechanism, which has been shown to modulate reasoning in other contexts, was not available in our competitive condition.

Our results suggest that children’s sensitivity to group competition as reflected in their reasoning depended on the kind of game they played. That is, when children played the dollhouse game, they produced more reasons and transacts in the competitive condition than in the non-competitive condition; whereas when they played the zoo game, they produced more reasons and more transacts in the non-competitive condition than in the competitive condition. The exact same pattern was observed in each age group, although 7-year-olds produced more reasons and transacts overall than did 5-year-olds. Thus, our findings suggest that group competition did not influence children’s collaborative reasoning consistently across different contexts.

One explanation for this could be that children might be less familiar with the zoo set-up as compared to the house set-up. Thus, in the non-competitive condition, children might have produced more reasons and transacts to explore various imaginary possibilities (e.g., having a dog in a zoo), when playing the zoo game than when playing the house. Nevertheless, a theoretical motivation for why some topics should lend themselves specifically to discussions under outgroup competition or under heightened motivation, as compared to when such a motivation is absent, is outstanding.

We observed an age effect as expected and common in this age range: children produce reasons more frequently in their discourse with age [11–13,20,30]. However, prior research found that 7-year-olds are more sensitive to competition than 5-year-olds [20]. Yet, in our study, 5- and 7-year-old children responded to group competition or to the conditions the same way (only 7-year-olds provided more reasons overall) probably because both conditions were collaborative.

To conclude, a widespread intuition is that rivalry fosters excellence, be it in sports competitions or lay conceptions of market economy. But it is important to look closely at what exactly it is about a competitive situation that influences action—and what kinds of action. In the present study, we looked specifically at the production of reasons in the mere presence of competition as a motivational cue. This stimulus has not been sufficient to trigger a robust improvement in reasoning. Future studies could use more lively cues for group competition such as running two actually competing teams side by side, which could create a sufficient contrast between the two contexts of collaborative reasoning.

## Supporting information

S1 Table Model summaries. Fixed effects of the main effects model and the model containing the game by condition interaction.
(PDF)

S1 File Materials. Stimuli used in the experiment.
(DOCX)

S2 File Test protocol. Description of the procedure and instructions.
(DOCX)

S3 File Data. CSV-format data summarized at the per-trial level.
(CSV)

S4 File Data analysis and plotting code in R Markdown. Reproduces all analyses and plots using the dataset in S3 File.
(RMD)
