# Supplementary material for: Collaborative reasoning in the context of group competition
Source: PLoS One. 2021 Feb 5;16(2):e0246589. doi: 10.1371/journal.pone.0246589 (PMC7864449; doi:10.1371/journal.pone.0246589)
Supplement: S1 Table — Fixed effects of the main effects model and the model containing the game by condition interaction. (PDF) [file pone.0246589.s001.pdf]

S3 - Model parameters reasons

Table S3 (a): Fitted parameter values of the GLM containing the main effects of age group, condition and game, and the control variables of gender and trial.
Model formula:

```
glmer(reasons ~ condition + ageGroup +
                game + gender + trial + (1|dyad) +
                offset(log(utterances)) )
```

	Estimate	SE	z value	Pr(>|z|)	
1. Intercept	-2.462	.141	-17.417	<.001	***
2. Age group (7 years)	.448	.138	3.243	.001	**
3. Condition (Non-competitive)	.109	.078	1.393	.164	
4. Game (Zoo)	-.116	.078	-1.484	.138	
5. Gender (Male)	-.082	.136	-.605	.545	
6. Trial (Second)	-.035	.075	-.472	.637	

Table S3 (b): Fitted parameter values for the GLM containing the interaction between condition and game along with the main effects of age group and condition and the control variables of gender and trial.
Model formula:

```
glmer(reasons ~ condition * game + ageGroup +
                gender + trial + (1|dyad) +
                offset(log(utterances)) )
```

	Estimate	SE	z value	Pr(>|z|)	
1. Intercept	-2.296	.141	-16.277	<.001	***
2. Age group (7 years)	.443	.128	3.468	<.001	***
3. Condition (Non-competitive)	-.226	.143	-1.578	.115	
4. Game (Zoo)	-.483	.156	-3.098	.002	**
5. Gender (Male)	-.067	.125	-.536	.592	
6. Trial (Second)	-.033	.075	-.438	.661	
7. Interaction Condition : Game	.702	.254	2.762	.006	**

S3 - Model parameters transacts

Table S3 (c): Fitted parameter values of the GLM containing the main effects of age group, condition and game, and the control variables of gender and trial.
Model formula:

```
glmer(transacts ~ condition + ageGroup +
                  game + gender + trial + (1|dyad) )
```

	Estimate	SE	z value	Pr(>|z|)	
1. Intercept	1.809	.119	15.155	< 2e-16	***
2. Age group (7 years)	.408	.118	3.445	< .001	***
3. Condition (Non-competitive)	.033	.071	.465	.642	
4. Game (Zoo)	-.195	.071	-2.753	.006	**
5. Gender (Male)	-.055	.117	-.471	.638	
6. Trial (Second)	-.324	.071	-4.562	<.001	***

Table S3 (d): Fitted parameter values for the GLM containing the interaction between condition and game along with the main effects of age group and condition and the control variables of gender and trial.
Model formula:

```
glmer(transacts ~ condition * game + ageGroup +
                  gender + trial + (1|dyad) )
```

	Estimate	SE	z value	Pr(>|z|)	
1. Intercept	1.952	.124	15.782	< 2e-16	***
2. Age group (7 years)	.405	.112	3.609	<.001	***
3. Condition (Non-competitive)	-.252	.129	-1.960	.050	*
4. Game (Zoo)	-.496	.134	-3.701	<.001	***
5. Gender (Male)	-.049	.111	-.437	.662	
6. Trial (Second)	-.324	.071	-4.553	<.001	***
7. Interaction Condition : Game	.595	.223	2.663	.008	**
