# Supplementary material for: Collaborative reasoning in the context of group competition
Source: PLoS One. 2021 Feb 5;16(2):e0246589. doi: 10.1371/journal.pone.0246589 (PMC7864449; doi:10.1371/journal.pone.0246589)

Materials Used

Cooperative Reasoning in the Context of Group Competition

# Warm-up

## Background picture

A picnic basket against a background of grass, 5 circles the size of the items used placed on the basket

## Items

### Default items

purple drinking bottle, white picnic blanket, torchlight, scissors, red drinking bottle

### Child A inadequate items

teddy bear, alarm clock, rubber duck, broken umbrella, diving goggles

### Child A adequate items

pineapple, watermelon, red picnic blanket, apples, tart

### Child B inadequate items

alarm clock, teddy bear, slingshot, rope ladder, brush and pallet

### Child B adequate items

pretzel, cutlery, purple drinking bottle, muffin, bananas

# Zoo game

## Background picture

A 4-color graphical birds-eye view zoo map, 7 circles the size of the items used placed around the map

## Items

### Default items

empty meadow, vulture, donkey, goats, shark, shark, meerkats

### Child A inadequate items

dog, slug, pigeon, fly, mouse, worm, mole

### Child A **adequate** items

elephants #1, lions #1, camel #1, chimps #1, zebras, bears, penguins

### Child B **inadequate** items

sparrow, snail, pigeon, beetle, wasp, spider, mole

### Child B **adequate** items

elephants #2, lions #2, dromedaries, chimps #2, rhinoceros, giraffes, kangaroos

# Dollhouse game

## Background picture

A dollhouse with 9 rooms, 7 circles the size of the items used placed in 7 of these rooms

## Items:

### Default items

TV set #1, chest, toilet, hanging lamp, TV set #2, refrigerator, withered plant

### Child A inadequate items

deer, tree, bicycle, excavator, binoculars, locomotive, diving goggles

### Child A adequate items

bathtub #1, stove #1, armchair #1, bed #1, table, wardrobe, dishes

### Child B inadequate items

boar, crane, rubber boat, sailing boat, airplane, mushrooms, football

### Child B adequate items

bathtub #2, stove #2, armchair #2, bed #2, chandelier, chairs, rug

# Token trays:


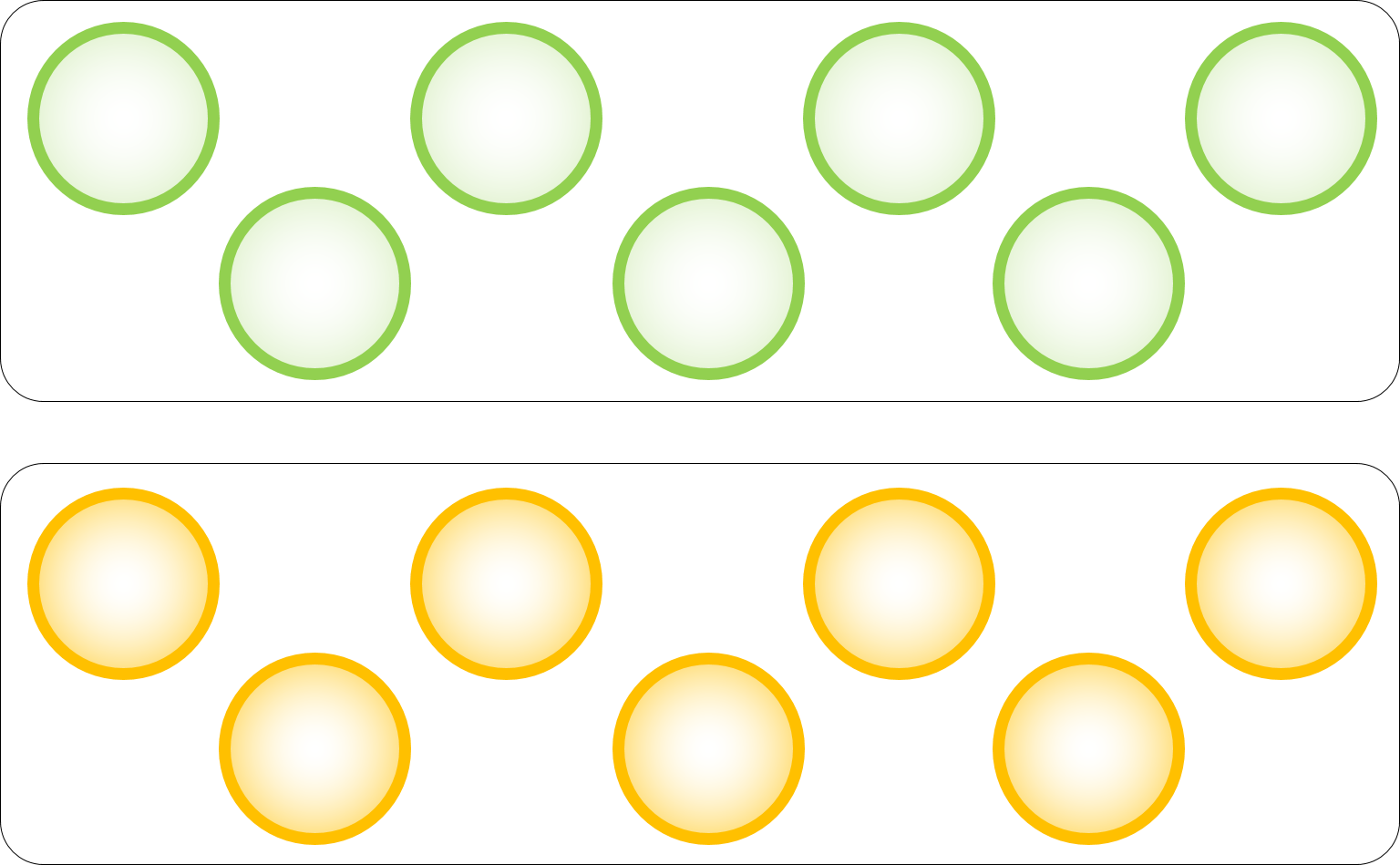

Supplement: S1 File — Stimuli used in the experiment. (DOCX) [file pone.0246589.s002.docx]
