# Supplementary material for: Collaborative reasoning in the context of group competition
Source: PLoS One. 2021 Feb 5;16(2):e0246589. doi: 10.1371/journal.pone.0246589 (PMC7864449; doi:10.1371/journal.pone.0246589)
Supplement: S2 File — Description of the procedure and instructions. (DOCX) [file pone.0246589.s003.docx]

Procedure

Cooperation under outgroup competition

During the warm-up phase, the procedure is guided by the goal of children’s familiarization with the materials and type of task that they are facing. It is not characterized by verbatim communication of standardized verbal material, although conversations did turn out highly similar given the constant experimental context. During the final instructions in each test trial the experimenter adheres to an exact text.

The procedure is described below with the example of the dollhouse game coming first and the zoo game in the competitive condition. Both variables were counterbalanced in the study. The procedure took place in German.

# Warm-up phase

- Children (C) each get a yellow baseball cap and the experimenter (E) refers to both as the yellow team.
- E asks C whether they like to do picnics and what things are needed.
- E gives each C ten items (labelled Child A and B in Appendix S1) and asks them to select five each that go well with a picnic, and discard the others in a box.
- E produces the warm-up background picture (a picnic basket) with five default items (tokens already placed on the five slots in the picture), commenting that it will need to be fixed, entering into the following loop:
  - C are asked to identify one default item that they want to discard, and to produce a reason why. Once a satisfactory reason has been produced, E concurs and C are asked to discard that item in the box.
  - C are asked to select an alternative item from their joint prior selection to put into the basket, and to give a good reason, receiving praise for fulfilment.
  - C are asked whether there is something left they want to fix, repeating the loop in the affirmative case.
- Warm-up materials are removed under praise for C’s good job.

The purpose of the prior individual selection of items ahead of each trial is to raise the motivation of replacing items in the game by giving children’s own items the status of being superior relative to the other, discarded, items. Also, this way, we ensure that children have inspected and are aware of the content of their tokens. Furthermore, duplicate items are distributed across children’s individual item sets to make available first easy grounds for arguing for and against some items during the discussion of item placements in each trial.

# Test trials

## Dollhouse game

- E puts a token tray in front of both C and asks whether C know about dollhouses and what things can be found in them.
- E puts a barrier between C, so each does the following token selection privately.
- E gives C the 14 dollhouse tokens labelled Child A and B in Appendix S1, instructing to select those seven and place them on their tray that C would rather put into a dollhouse, and to discard the others in the box.
- E removes the barrier and merges the resulting selection of 14 items into one heap, removing the trays.
- E produces the dollhouse background with default items, commenting that it will need to be fixed.

### Instruction

“You don’t start before I’m out. Here is your task: You now build a really nice dollhouse together, one in which dolls would love to live, and discuss together what things you would like to replace for that. But remember that each time, you need to say a good reason for why you want to replace it.”

#### Condition manipulation

E opens the laptop, commenting, “Let’s see if you are playing the dollhouse game against someone else.”

#### *Non-competitive*

“No, you are playing the dollhouse game alone, without opponents.” E shuts the laptop and places it outside children’s playing space.

#### Concluding instruction

“Now remember, build a really nice dollhouse together, one that’s good to live in, and discuss what things you need to replace for that. But remember each time to say a really good reason for why it is you want to replace something. I’m now going out, and once you are ready, you come and call me back in, okay?”

## Zoo game

- E puts a token tray in front of both C and asks whether C know about zoos and what animals can be found in them.
- E puts a barrier between C, so each does the following token selection privately.
- E gives C the 14 zoo tokens labelled Child A and B in Appendix S1, instructing to select those seven and place them on their tray that C would rather expect in a zoo, and to discard the others in the box.
- E removes the barrier and merges the resulting selection of 14 items into one heap, removing the trays.
- E produces the zoo background with default items, commenting that it will need to be fixed.

### Instruction

“You don’t start before I’m out. Here is your task: You now build a really nice zoo together, one that people would love to visit, and discuss together what things you would like to replace for that. But remember that each time, you need to say a good reason for why you want to replace it.”

#### Condition manipulation

E opens the laptop, commenting, “Let’s see if you are playing the zoo game against someone else.”

#### ***Competitive***

“Oh yes, look, here’s the red team. The two kids from the red team have played the zoo game already. At the end we took this picture, but of course we can’t see what animals they chose.” The laptop image shows a gender-matched pair of children with red caps in front of the relevant background image with tokens masked. E places the open laptop in C’s view.

#### Concluding instruction

“Now remember, build a really nice zoo that people would love to visit, and discuss what animals need to be replaced for that. But remember each time to say a really good reason for why it is you want to replace animals. And if at the end you have a nicer zoo than the red team, then you win against them. I’m now going out, and once you are ready, you come and call me back in, okay?”
